# Risk factors for hospital readmission following complicated urinary tract infection

**DOI:** 10.1038/s41598-021-86246-7

**Published:** 2021-03-25

**Authors:** Tanya Babich, Noa Eliakim-Raz, Adi Turjeman, Miquel Pujol, Jordi Carratalà, Evelyn Shaw, Aina Gomila Grange, Cuong Vuong, Ibironke Addy, Irith Wiegand, Sally Grier, Alasdair MacGowan, Christiane Vank, Leo van den Heuvel, Leonard Leibovici

**Affiliations:** 1 Department of Medicine E, Beilinson Hospital, Rabin Medical Center, Petah-Tiqva, Israel; 2 Sackler Faculty of Medicine, Tel-Aviv University, Tel-Aviv, Israel; 3 Department of Infectious Diseases, Hospital Universitari de Bellvitge, Institut D’Investigació Biomèdica de Bellvitge (IDIBELL), Spanish Network for Research in Infectious Diseases (REIPI RD12/0015), Instituto de Salud Carlos III (ISCIII), Madrid, Spain; 4 Department of Infectious Diseases, Hospital Universitari Parc Taulí, Barcelona, Spain; 5 AiCuris Anti-Infective Cures GmbH, Wuppertal, Germany; 6 Department of Infection Sciences, Southmead Hospital, North Bristol NHS Trust, Bristol, UK; 7 Julius Center for Health Sciences and Primary Care, University Medical Center Utrecht, Utrecht, The Netherlands

**Keywords:** Diseases, Medical research, Risk factors

## Abstract

Hospital readmissions following severe infections are a major economic burden on the health care system and have a negative influence on patients' quality of life. Understanding the risk factors for readmission, particularly the extent to which they could be prevented, is of a great importance. In this study we evaluated potentially preventable risk factors for 60-day readmission in patients surviving hospitalization for complicated urinary tract infection (cUTI). This was a multinational, multicentre retrospective cohort study conducted in Europe and the Middle East. Our cohort included survivors of hospitalization due to cUTI during the years 2013–2014. The primary outcome was 60-day readmission following index hospitalization. Patient characteristics that could have influenced readmission: demographics, infection presentation and management, microbiological and clinical data; were collected via computerized medical records from infection onset up to 60 days after hospital discharge. Overall, 742 patients were included. The cohort median age was 68 years (interquartile range, (IQR) 55–80) and 43.3% (321/742) of patients were males. The all-cause 60-day readmission rate was 20.1% (149/742) and more than half were readmitted for infection [57.1%, (80/140)]. Recurrent cUTI was the most frequent cause for readmission [46.4% (65/140)]. Statistically significant risk factors associated with 60-day readmission in multivariable analysis were: older age (odds ratio (OR) 1.02 for an one-year increment, confidence interval (CI) 1.005–1.03), diabetes mellitus (OR 1.63, 95% CI 1.04–2.55), cancer (OR 1.7, 95% CI 1.05–2.77), previous urinary tract infection (UTI) in the last year (OR 1.8, 95% CI: 1.14–2.83), insertion of an indwelling bladder catheter (OR 1.62, 95% CI 1.07–2.45) and insertion of percutaneous nephrostomy (OR 3.68, 95% CI 1.67–8.13). In conclusion, patients surviving hospitalization for cUTI are frequently re-hospitalized, mostly for recurrent urinary infections associated with a medical condition that necessitated urinary interventions. Interventions to avoid re-admissions should target these patients.

## Introduction

Patients surviving hospitalization are frequently readmitted. About 20% of patients are re-hospitalized during the first month after discharge. These readmissions have a vast implication by negatively influencing the patients' quality of life and imposing a significant economic burden on the health care system ^1^.

Severe sepsis survivors are particularly prone to readmission, and up to 50% are readmitted within 6 months of discharge ^2^. Global initiatives focusing on early diagnosis and proper management to improve the survival of patients hospitalized with severe sepsis, had led to a decrease of in-hospital mortality rates. As a result, the focus has shifted to understanding the survivors' rehabilitation process and reducing preventable readmissions ^3–5^. Recent studies addressing this issue have demonstrated that a prolonged length of hospital stay during the index hospitalization was associated with higher rates of readmission. Also, patients discharged to care facilities had a higher risk for readmission compared to patients who were discharged home ^2,6,7^.

Complicated urinary tract infection is a common nosocomial infection that is responsible for a major share of hospital admissions, and account for 20% to 40% of severe sepsis cases ^8,9^. These infections are associated with catheterization and anatomical or functional modification of the urinary tract and thus a subject to frequent re-hospitalizations ^10^. Understanding the avoidable risk factors for readmission may inform policy for optimal care during hospitalization and proper post-discharge ambulatory care.

Therefore, we aimed to determine potentially preventable risk factors for readmission in patients surviving hospitalization following complicated urinary tract infection.

## Methods

### Study design, setting, and participants

This was a multinational, multicenter, retrospective cohort study that involved collection of data on hospitalized patients, diagnosed with complicated urinary tract infection (cUTI) as a primary cause of hospitalization or developed cUTI throughout hospitalization between 1st January 2013 to 31st December 2014. The study was carried out in 20 centers (8 countries) around Europe and the Middle East (eTable1). Patients were identified by systematic screening via hospital administration system ^11^ for CD-9 CM or ICD-10 CM Codes at discharge ^12,13^. Eligible patients were reviewed for inclusion at each site.

This cohort was previously described in a paper by Elikaim-Raz and et al. ^14^.

Inclusion criteria were according to adapted Food and Drug Administration (FDA) guidance, European medicine agency (EMA) and clinical practice guidelines on definition of cUTI's: Adult patients with UTI and at least one of the following: neurogenic bladder, indwelling urinary catheter, renal transplantation, pyelonephritis with normal urinary tract anatomy, obstructive uropathy, renal impairment caused by intrinsic renal disease, urinary retention or urinary tract modifications; and at least one of the following signs or symptoms: dysuria, urinary frequency, or urinary urgency, UTI-related altered mental state, chills or rigors associated with fever or hypothermia, costo-vertebral angle tenderness on physical examination, flank pain or pelvic pain and bacteriuria of 10^5^ CFU/mL or greater of no more than 2 pathogens in urine culture or a positive blood culture growing possible uropathogens (of maximum 2 pathogens) without an alternative site of infection. In the present analysis we included patients that did not acquire their infection in the hospital, and patients that their index hospitalization did not result in death.

Exclusion criteria included: prostatitis, polymicrobial infections including Candida spp., polymicrobial infections of more than 2 pathogens and cUTI with Candida spp. as the only growth.

### Definitions and outcomes

Data were extracted from computerized medical records and hospital administration system into a web-based electronic case report form (eCRF). Automatic validation programs, monitoring, and audit were conducted by a designated third party.

Data were collected from infection onset up to 60 days after hospital discharge: demographics, patient characteristics, comorbidities, Charlson comorbidity index ^15^, characteristics and presentation of infection, microbiological and clinical data, clinical management of infection including empirical antibiotic treatment, outcomes and details on readmission including reason for readmission and length of hospital stay. Cancer was defined as one of the following: leukemia, lymphoma, solid tumor or metastatic disease. Pyelonephritis was defined as a urinary tract infection with normal urinary tract anatomy, in the presence of fever, and involvement of the kidney as evidenced by flank pain or tenderness or findings on imaging.

Our primary outcome was readmission to the hospital during 60 days following index hospitalization.

### Ethics statements

The study was approved by the institutional review board at Rabin Medical Center, Compus Beilinson and at each participating site in accordance with local regulatory requirements. Data were retrieved and handled anonymously in accordance with the local data protection regulation and European Directive on the Privacy of Data (EU (95/46/EC) ^16^. Also, all methods were carried out in accordance with relevant guidelines and regulations. The committees waived the need for informed consent.

### Statistical analysis

IBM SPSS statistics 25 software was used for statistical analysis. *P* values are two-tailed and *P* < 0.05 was considered statistically significant.

For the univariate analysis the following tests were used: for categorical variables- Chi-square test or Fisher's exact test as appropriate, for continuous variables—Mann–Whitney test or student's t test if normally distributed.

Statistically significant variables were tested for multicollinearity by a correlation matrix and variance inflation factor (VIF) to determine the degree of correlation.

To identify independent risk factors for hospital readmission multivariable analysis using generalized estimating equation (GEE) binary logistics (to account for study site as a random effect variable) was performed. Statistically significant variables found in univariate analysis, not strongly correlated and clinically relevant where introduced in this model. The Quasi-likelihood under the independence model criterion (QIC) helped to adjust the best model.

### Prior presentations

Due to COVID-19 the presentation of this article at the 30th ECCMID (European Congress of Clinical Microbiology and Infectious Disease was cancelled.

## Results

### Patient characteristics

We analyzed 742 patients. Demographics, patient characteristics, infection presentation and management of the index hospitalization are presented in Tables 1, 2 and 3. Among the study cohort, 43.3% (321/742) of patients were males and the median age was 68 years (IQR 55–80). Approximately 30% of patients had recurrent UTI infections during the last year. Fourteen percent resided in long term care facilities (107/742) and were dependent on others for daily activities (105/742) (Table 1). Bacteremia during the index hospitalization was documented in 18.5% (147/742) of patients (Table 2). The median length of hospital stay during the index hospitalization was 7 (IQR 4.75–11) and the majority of patients (87.9%, 652/742) were discharged home (Table 3).

**Table 1:** Patient characteristics for 60-day readmission.

	Entire cohort N = 742	60-day readmission (yes) N = 149	60-day readmission (no) N = 593	*P* value
Gender ( male)	321 (43.3%)	85 (57%)	236 (39.8%)	0.000
Age (median, (IQR))	68 (55–80)	73 (64.5–83)	67 (53–79)	0.000
Previous 30 day antibiotic treatment	146/741 (19.7%)	40 (26.8%)	106/592 (17.9%)	0.014
Place of residence—LTCF	107 (14.4%)	32 (21.5%)	75 (12.6%)	0.006
Functional capacity-Depended/bed ridden	105/740 (14.2%)	33 (22.1%)	72/591 (12.2%)	0.002
Previous UTI infection in the last year	206/741 (27.8%)	57 (38.3%)	149/592 (25.2%)	0.001
Indwelling catheter at diagnosis	219/740 (29.6%)	59 (39.6%)	160/591 (27.1%)	0.003
Urinary retention	158/741 (21.3%)	43 (28.9%)	115/592 (19.4%)	0.012
Neurogenic bladder	35/740 (4.7%)	12/148 (8.1%)	23 (3.9%)	0.03
Renal impairment	191 (25.7%)	40 (26.8%)	151 (25.5%)	0.73
Obstructive uropathy	170/740 (23%)	36 (24.2%)	134/591 (22.7%)	0.7
Urinary tract modification	93/738 (12.6%)	29 (19.5%)	64/589 (10.9%)	0.005
Immunosuppressive theraphy	76 (10.2%)	16 (10.7%)	60 (10.1%)	0.823
Corticosteroid therapy	44 (5.9%)	8 (5.4%)	36 (6.1%)	0.746
**Comorbidities**				
Dementia	102 (13.8%)	32 (21.5%)	70 (11.8%)	0.002
Myocardial infection	50 (6.7%)	14 (9.4%)	36 (6.1%)	0.148
Heart failure	129 (17.4%)	25 (16.8%)	104 (17.5%)	0.827
Peripheral vascular disease	57 (7.7%)	15 (10.1%)	42 (7.1%)	0.221
Cerebrovascular disease	132 (17.8%)	32 (21.5%)	100 (16.9%)	0.188
Chronic pulmonary disease	97 (13.1%)	19 (12.8%)	78 (13.2%)	0.897
Ulcer disease	28 (3.8%)	5 (3.4%)	23 (3.9%)	0.765
Diabetes	191 (25.7%)	53 (35.6%)	138 (23.3%)	0.002
Chronic kidney disease	212 (28.6%)	53 (35.6%)	159 (26.8%)	0.034
Hemiplegia	61 (8.2%)	15 (10.1%)	46 (7.8%)	0.359
Liver disease	42 (5.7%)	8 (5.4%)	34 (5.7%)	0.863
Solid tumor/ homological malignancies (cancer)	120 (16.2%)	39 (26.2%)	81 (13.7%)	0.000
Active chemotherapy	20 (2.7%)	9 (6%)	11 (1.9%)	0.005
Charlson score (median, (IQR))	2 (0–4)	3 (1–5)	2 (0–4)	0.000

Abbreviations: UTI, urinary tract infection; LTCF, long term care Facility;

**Table 2:** Infection presentation.

	Entire cohort N = 742	60-day readmission (yes) N = 149	60-day readmission (no) N = 593	*P* value
Pyelonephritis	183 (24.7%)	19 (12.8%)	164 (27.7%)	0.000
Septic shock	19/688 (2.8%)	4/135 (3%)	15/553 (2.7%)	0.879
Bacteremia on sepsis onset (the first 48 h)	147 (18.5%)	36 (23.4%)	111 (17.3%)	0.096
ESBL-pathogen	139 (18.7%)	35 (23.5%)	104(17.5%)	0.639
ICU admission	51 (6.9%)	14 (9.4%)	37 (6.2%)	0.173
mechanical ventilation	29 (3.9%)	8 (5.4%)	21 (3.5%)	0.303
Pseudomonas aeruginosa infection	54/738 (7.3%)	15/148 (10.1%)	39/590 (6.6%)	0.141
E.coli infection	433 (58.4%)	66 (44.3%)	367(61.9%)	0.000

Abbreviations: ESBL, Extended-spectrum beta-lactamases; ICU, intensive care unit.

**Table 3:** Infection management.

	Entire cohort N = 742	60-day readmission (yes) N = 149	60-day readmission (no) N = 593	*P* value
Appropriate empirical treatment	348/613 (56.8%)	72/123 (58.5%)	276/520 (56.3%)	0.658
Insertion of indwelling bladder catheter	266/735 (36.2%)	68/148 (45.9%)	198/587 (33.7%)	0.006
insertion of percutaneous nephrostomy	28 (3.8%)	14 (9.4%)	14 (2.4%)	0.000
Indwelling catheter replacement treatment	93/736(12.6%)	30 (20.1%)	63/587 (10.7%)	0.002
Renal replacement therapy	18 (2.4%)	4 (2.7%)	14 ( 2.4%)	0.818
Antibiotic adverse events	43/739 (5.8%)	13/148 (8.8%)	30/591 (5.1%)	0.085
Length of antibiotic treatment (days) (median, (IQR))	6 (4–9)	6 (4–11)	6 (4–9)	0.117
Length of hospital stay (days) (median, (IQR))	7 (4.75–11)	8 (5–13)	7 (4–11)	0.021
Discharge- home	652 (87.9%)	117 (78.5%)	535 (90.2%)	0.000
Antibiotic treatment prescribed at discharge	392/685 (57.2%)	79/142 (55.6%)	313/543 (57.6%)	0.667
Length of antibiotic treatment prescribed at discharge (days) (median, (IQR)), N = 679	4 (0–7)	4 (0–7)	5 (0–7)	0.701
*Length of antibiotic treatment prescribed at discharge (days) (median, (IQR)), N = 386	7 (5–10)	7 (5–10)	7 (5–10)	0.606

*Among patients with prescribed antibiotics at discharge.

### Readmission

The all-cause 60-day readmission rate was 20.1% (149/742). More than half were readmitted for infection [57.1%, (80/140)]. The most common cause for readmission was recurrent cUTI [46.4% (65/140)]. A quarter of non-infection related readmissions were for urinary tract abnormalities or instrumentation (eTable2). The readmission median length of hospital stay was 7 days (IQR 4–12). Readmission rates per participating country and center are stated in eTable3 and eTable4.

### Patient-related risk factors

Readmitted patients were significantly older compared to non-readmitted patients 73 years (IQR 64.5–83) vs 67 years (IQR 53–79) and were more likely to be male: 57% (85/149) vs 39.8% (236/593). Comorbidities such as heart failure, diabetes, chronic kidney disease and cancer were more prevalent among the readmitted group then the comparator group. This was also demonstrated by the Charlson comorbidity score.

The same was true for functional decline, residency in long term facilities and previous UTI infection during the last year, all more prevalent among 60-day readmission compared to those without [Table 1]. Patients admitted with indwelling urinary catheter (index hospitalization) were more likely to require hospital readmission 41/149 (27.5%) vs 114/591 (19.3%) for non-readmission, *P* = 0.027. Similarly, patients with urinary retention: 28.9% (43/149) vs 19.4% (115/592); neurogenic bladder: 8.1% (12/148) vs 3.9% (23/593); and urinary tract modification: 19.5% (29/149) vs 10.9% (64/589) were more prone to readmission (Table 1).

### Infection-related risk factors

Sepsis severity (septic shock, bacteremia, intensive care unit (ICU) admission and mechanical ventilation) did not differ between patients that were readmitted to those who were not. Patients hospitalized with pyelonephritis were less likely to be readmitted during 60 days of discharge [12.8% (19/149) vs 27.7% (164/593), *P* = 0.000] (Table 2).

### Management-related risk factors

Urological interventions such as indwelling catheter replacement treatment and percutaneous nephrostomy were significantly more frequent among readmitted patients compared to those who were not [20.1% (30/149) vs 10.7% (63/587)] and [9.4% (14/149) vs 2.4% (14/593), respectively]. Patients requiring readmission had a significantly longer length of index hospital stay [8 days (IQR 5–13)], in comparison to patients not readmitted [7 days (IQR 4–11), *P* = 0.021] and they were less likely to be discharged home [78.5% (117/149) vs. 90.2% (535/593), *P* = 0.000] (Table 3). We found no difference in the duration of the antibiotic treatment during hospital stay and afterwards.

### Multivariable analysis

Multivariable analysis revealed the following risk factors as statistically associated with 60-day readmission: older age (OR 1.02 for a one-year increment, CI 1.005–1.03), cancer (OR 1.7, 95% CI 1.05–2.77), diabetes mellitus (OR 1.63, 95% CI 1.04–2.55), previous UTI infection in the last year (OR 1.8, 95% CI: 1.14–2.83), insertion of an indwelling bladder catheter (OR 1.62, 95% CI 1.07–2.45) and insertion of percutaneous nephrostomy (OR 3.68, 95% CI 1.67–8.13) (Table 4).

**Table 4:** Multivariable analysis for 60-day readmission.

Risk factor	Multivariable logistic regression analysis OR (95% CI)	Univariate analysis OR (95% CI)
Year of age	1.02 (1.005–1.03)*	1.02 (1.01–1.04)
Previous 30 day antibiotic treatment	1.37 (0.84–2.25)	1.68 (1.11–2.56)
Previous UTI infection in the last year	1.8 (1.14–2.83)*	1.84 (1.26–2.69)
Neurogenic bladder	2.15 (0.97–4.76)	2.18 (1.06–4.96)
Urinary tract modification	1.64 (0.92–2.95)	1.98 (1.22–3.21)
Dementia	1.47 (0.82–2.63)	2.04 (1.28–3.24)
Cancer	1.7 (1.05–2.77)*	2.24 (1.45–3.46)
Diabetes mellitus	1.63 (1.04–2.55)*	1.66 (1.13–2.42)
Chronic kidney disease	1.12 (0.73–1.74)	1.51 (1.03–2.21)
Pyelonephritis	0.58 (0.33–1.04)	0.38 (0.23–0.64)
Insertion of an indwelling bladder catheter	1.62 (1.07–2.45)*	1.67 (1.16–2.41)
indwelling bladder catheter–replacement therapy	1.54 (0.91–2.61)	2.1 (1.3–3.38)
Insertion of percutaneous nephrostomy	3.68 (1.67–8.13)*	4.29 (1.99–9.21)
Length of hospital stay	1.003 (0.99–1.01)	1.004 (1.001–1.01)
Discharge- home	0.63 (0.35–1.13)	0.39 (0.25–0.64)

Risk factors for 60-day readmission, univariate and multivariate logistic regression analysis, goodness of fit -Quasi likelihood under independence model criteria (QIC) − 674.3, N = 728, constant β =  − 1.701.

Age-per one-year increment; Length of hospital stay: per 1-day increment.

*Statistically significant *p* < 0.05.

## Discussion

In this large multinational cohort one out of five patients hospitalized for cUTI was readmitted during the following 60 days after discharge. Among them, in nearly 40% the reason was recurrent cUTI. We found that previous UTI infection in the last year prior the index hospitalization and insertion of urological devices (indwelling bladder catheter or percutaneous nephrostomy) as a treatment strategy for cUTI were strong predictors of 60-day readmission. These findings suggest that anatomic abnormalities /dysfunction of the urinary tract are major risk factors for readmission. The rates of readmission were lower in patients with pyelonephritis. This can be explained by the fact that these patients are usually young females with a normal urinary tract anatomy and as such have a better prognosis compared to patients with other reasons for cUTI ^17^.

Disappointingly neither appropriate empirical antibiotic treatment nor the duration of antibiotic treatment had an influence on readmission. Prescott H.C et al. demonstrated that infection related readmissions compared to other medical conditions within 90-day discharge following sever sepsis can be partially prevented by early treatment and a better follow-up care ^18^. Quality of care during the index hospitalization is of importance for reducing readmissions following sever sepsis. the surviving sepsis campaign guidelines stress the need for early identification of infection and proper early antibiotic treatment ^5^.

Additional significant predictors for 60-day readmission were older age and comorbidities such as diabetes mellitus and cancer. Length of hospital stay and discharge to long term facilities were not statistically associated with readmission in the multivariable analysis.

In our study, similarly to previously published studies, the majority of readmissions are due to recurrent infection among sepsis survivals ^6,19,20^. Sun et al.^19^ retrospectively reviewed 444 sepsis survivals at risk for readmission from three acute care hospitals. The overall readmission rate was 23.4% with infection being the most common reason for hospital readmission. Among them, 51.4% were recurrent/unresolved infections. DeMerle et al.^20^ conducted a retrospective study on 1,588 survivals of hospitalization for sepsis to identify the extent to which the readmission was due to same site and pathogen. They found that among 472 patients re-hospitalized 29.1% were for sepsis, of them 68.6% were for the same site but only one fifth were culture positive. Also, the most common site of infection was the urinary tract (29.2%).

Several studies were performed in order to identify risk factors for hospital readmission among sepsis survivals. Goodwin et al.^2^ retrospectively analyzed severe sepsis survivals from three hospitals around U.S states to evaluate predictors for hospital readmission. Among the 43,425 survivals, 20,907 patients were readmitted during 180 days. Significant risk factors were age, male gender, race, insurance status, comorbidities such as malignancy, diabetes lung and chronic kidney disease, longer length of stay and discharge to care facilities.

To the best of our knowledge our study is the first to focus on patients with cUTI, hence the etiology of infection and predictors of readmission differ from previously published studies. Our study suggests that the presence of underlying disease which accompanies these patients, and especially pathologies and instrumentation of the urinary tract predisposes them to recurrent infections and frequent hospital admissions. Post-discharge strategies and follow up care to reduce readmission among these patients should be implemented and tested. Extensive strategies such as arranged follow-ups, providing discharge summaries for community physicians and patient education to reduce readmissions for other conditions have been implemented and found useful in many studies ^21,22^. Although the need of our study papulation differ from previously published studies they provide evidence that the link between hospital caregivers and outpatient care may be effective in reducing readmissions.

Our study has several limitations. First, this was an observational retrospective study, and as such a relatively weak design and particularly prone to selection bias. Secondly, patients' were collected from 20 hospitals, in which the local management and diagnostic guidelines may differ. We tried to minimize this bias by close monitoring and strict adherence to the study protocol. Moreover, in the analysis, study center was added as a random variable, accounting for clustering by center. Third, we could not capture patients' readmitted to hospitals with a different administrative system. This may underestimate the rate of readmissions and hence weaken the association. We did not collect data on the pathogens of the urinary tract infections that caused readmission. Our study's strength lies in its large sample size, a multinational and multicenter design and the fact that it focuses on a specific group of patients with cUTI. These strengths encourage the use of our results for benchmarking.

Patients with complicated UTI and abnormalities or instrumentation of the urinary tract should be closely monitored after discharge from the hospital. Removal of catheters and drains as soon as possible in the community will probably prevent recurrent infections and re-admissions. Although we could not show a difference made by the duration of antibiotic treatment, further research on the need for antibiotic prophylaxis, the most suitable antibiotic drugs for acute episode and timing of device removal to prevent recurrences should be performed in these patients. The results of this study stress the necessity to examine re-admissions separately in groups of patients according to their main reason for hospital stay.

## Supplementary Information

Supplementary Information
