# Supplementary material for: Risk factors for hospital readmission following complicated urinary tract infection
Source: Sci Rep. 2021 Mar 25;11:6926. doi: 10.1038/s41598-021-86246-7 (PMC7994309; doi:10.1038/s41598-021-86246-7)
Supplement: Supplementary file 1 — Supplementary Information [file 41598_2021_86246_MOESM1_ESM.docx]

Risk factors for hospital readmission following complicated urinary tract infection

**Authors:** Tanya Babich ^1,2^M.Sc, Noa Eliakim-Raz ^1,2^MD, Adi Turjeman ^1,2^M.Sc, Miquel Pujol ^3^ MD, Jordi Carratalà ^3^MD, Evelyn Shaw ^3^MD, Aina Gomila Grange ^3,4^ MD, Cuong Vuong ^5^ PhD, Ibironke Addy ^5^PhD, Irith Wiegand ^5^ PhD, Sally Grier ^6^BSc, Alasdair MacGowan ^6^PhD, Christiane Vank ^5^PhD, Leo van den Heuvel ^7^ BA, Leonard Leibovici ^1,2^MD

**Affiliation:**

^1^Department of Medicine E, Beilinson Hospital, Rabin Medical Center, Petah-Tiqva, Israel

^2^Sackler Faculty of Medicine, Tel-Avv University, Israel

^3^Department of Infectious Diseases, Hospital Universitari de Bellvitge, Institut d'Investigació Biomèdica de Bellvitge (IDIBELL)), Spanish Network for Research in Infectious Diseases (REIPI RD12/0015), Instituto de Salud Carlos III (ISCIII), Madrid, Spain.

^4^Department of Infectious Diseases, Hospital Universitari Parc Taulí, Barcelona, Spain

^5^AiCuris Anti-infective Cures GmbH, Wuppertal, Germany.

^6^Department of Infection Sciences, Southmead Hospital, North Bristol NHS Trust, Bristol, UK.

^7^Julius Center for Health Sciences and Primary Care, University Medical Center Utrecht, Utrecht, the Netherlands.

**Running title:** Risk for hospital readmission

**Corresponding author:**

Mrs. Tanya Babitch, Medicine E, Beilinson hospital, Rabin Medical Center,Petah-Tikva, Israel. Phone number 972-527889816, Fax 937-3-9194665, Email: [tanyanoaba@clalit.org.il](mailto:tanyanoaba@clalit.org.il).

**Legend for tables - supplemental appendix online**

**eTable1**- List of participating centers

**eTable2-** Reasons for 60-day readmission

**eTable3-** Readmission within 60-days by participating countries

**eTable4-** Readmission within 60-days by participating centers

**eTable1:** List of participating centers

| **#** | **country** | **center** |
| --- | --- | --- |
| 1 | Bulgaria | Emergency Hospital Pirogov |
| 2 | Bulgaria | University Hospital Queen Joanna |
| 3 | Greece | Attikon University Hospital |
| 4 | Greece | Hippokration Hospital |
| 5 | Hungary | Kenezy Gyula Hospital |
| 6 | Hungary | Soproni Erzsébet Oktató Kórház és Rehabilitációs Intézet |
| 7 | Hungary | Szabolcs-Szatmár-Bereg Megyei Kórházak és Egyetemi Oktatókórház |
| 8 | Israel | Beilinson Hospital, Rabin Medical Center |
| 9 | Israel | Rambam Health Care Campus |
| 10 | Israel | Tel Aviv Medical Center |
| 11 | Italy | AORN dei Colli Monaldi |
| 12 | Italy | Azienda ospedaliero-universitaria policlinico di modena |
| 13 | Italy | National Institute for Infectious Diseases L. Spallanzani |
| 14 | Romania | Infectious Diseases Hospital Sfanta Parascheva Iasi |
| 15 | Romania | National Institute for Infectious Diseases "Prof Dr Matei Bals" |
| 16 | Spain | Bellvitge University Hospital |
| 17 | Spain | Hospital Universitario 12 de Octubre |
| 18 | Spain | Hospital Universitario Virgen Macarena |
| 19 | Turkey | Ankara Numune Egitim ve Araştırma Hastanesi |
| 20 | Turkey | Istanbul University Cerrahpasa Medical School |

**eTable2:** Reasons for 60-day readmission

Urinary tract dysfunction

N= 15/60 (25%)

Clostridium difficile

N=3/80 (3.7%)

Recurrent UTI

N=65/80 (81.2%)

\

Infection related readmission

N=80/140 (57.1%)

Non-infection related readmission

N= 60/140 (42.8%)

60-day readmission

N=149

**eTable3-** Readmission within 60-days by participating countries

| **Participating countries** | **readmission within 60-days** | **P value** |
| --- | --- | --- |
| Bulgaria | 5/54 (9.3%) | 0.000 |
| Greece | 8/68 (11.8%) |  |
| Hungary | 22/79 (27.8%) |  |
| Israel | 63/166 (38%) |  |
| Italy | 6/77 (7.8%) |  |
| Romania | 12/128 (9.4%) |  |
| Turkey | 5/58 (8.6%) |  |
| Spain | 28/112 (25%) |  |

**eTable4-** Readmission within 60-days by participating centers

| **Participating centers** | **readmission within 60-days** | **P value** |
| --- | --- | --- |
| Emergency Hospital Pirogov | 0 | 0.000 |
| University Hospital Queen Joanna | 5/34 (14.7%) |  |
| Attikon University Hospital | 4/21 (19%) |  |
| Hippokration Hospital | 4/47 (8.5%) |  |
| Kenezy Gyula Hospital | 3/17 (17.6%) |  |
| Soproni Erzsébet Oktató Kórház és Rehabilitációs Intézet | 7/28 (25%) |  |
| Szabolcs-Szatmár-Bereg Megyei Kórházak és Egyetemi Oktatókórház | 12/34 (35.3%) |  |
| Beilinson Hospital, Rabin Medical Center | 20/58 (34.5%) |  |
| Rambam Health Care Campus | 26/58 (44.8%) |  |
| Tel Aviv Medical Center | 17/50 (34%) |  |
| AORN dei Colli Monaldi | 0 |  |
| Azienda ospedaliero-universitaria policlinico di modena | 6/29 (20.7%) |  |
| National Institute for Infectious Diseases L. Spallanzani | 0 |  |
| Infectious Diseases Hospital Sfanta Parascheva Iasi | 7/71 (9.9%) |  |
| National Institute for Infectious Diseases "Prof Dr Matei Bals" | 5/57 (8.8%) |  |
| Bellvitge University Hospital | 11/53 (20.8%) |  |
| Hospital Universitario 12 de Octubre | 8/33 (24.2%) |  |
| Hospital Universitario Virgen Macarena | 9/26 (34.6%) |  |
| Ankara Numune Egitim ve Araştırma Hastanesi | 4/29 (13.8%) |  |
| Istanbul University Cerrahpasa Medical School | 1/29 (3.4%) |  |
